# 3D Bioprinting of In Vitro Models Using Hydrogel-Based Bioinks

**DOI:** 10.3390/polym13030366

**Published:** 2021-01-24

**Authors:** Yeong-Jin Choi, Honghyun Park, Dong-Heon Ha, Hui-Suk Yun, Hee-Gyeong Yi, Hyungseok Lee

**Affiliations:** 1 Department of Advanced Biomaterials Research, Korea Institute of Materials Science (KIMS), 797, Changwon 51508, Korea; jinchoi@kims.re.kr (Y.-J.C.); honghyun61@kims.re.kr (H.P.); yuni@kims.re.kr (H.-S.Y.); 2 EDmicBio Inc., Seoul 02445, Korea; matt@edmicbio.com; 3 Department of Rural and Biosystems Engineering, College of Agriculture and Life Sciences, Chonnam National University (CNU), Gwangju 61186, Korea; 4 Department of Mechanical and Biomedical Engineering, Kangwon National University (KNU), Chuncheon 24341, Korea; 5 Interdisciplinary Program in Biohealth-Machinery Convergence Engineering, Kangwon National University (KNU), Chuncheon 24341, Korea

**Keywords:** 3D bioprinting, in vitro model, hydrogel, bioink, 3D cell printing, tissue engineering

## Abstract

Coronavirus disease 2019 (COVID-19), which has recently emerged as a global pandemic, has caused a serious economic crisis due to the social disconnection and physical distancing in human society. To rapidly respond to the emergence of new diseases, a reliable in vitro model needs to be established expeditiously for the identification of appropriate therapeutic agents. Such models can be of great help in validating the pathological behavior of pathogens and therapeutic agents. Recently, in vitro models representing human organs and tissues and biological functions have been developed based on high-precision 3D bioprinting. In this paper, we delineate an in-depth assessment of the recently developed 3D bioprinting technology and bioinks. In particular, we discuss the latest achievements and future aspects of the use of 3D bioprinting for in vitro modeling.

## 1. Introduction

Severe acute respiratory syndrome coronavirus 2 (SARS-CoV-2)-associated coronavirus disease 2019 (COVID-19), has recently become a global pandemic that caused a serious economic crisis due to physical distancing and social disconnection [1]. Despite the urgent need to develop therapeutics for COVID-19 infection, no treatment has been developed to date due to a lack of sufficient knowledge about the emergence of new pathogens [1,2]. Moreover, as new pathogens (SARS-CoV, MERS-CoV) continue to emerge, appropriate models are needed to understand the disease pathology and to screen potential therapeutic agents efficiently [3]. Conventional models for drug validation mainly use animals. However, the development of animal models often involves long time periods, high costs, and are not useful for human applications due to species differences [4,5,6]. In addition, growing interest in animal ethics calls for alternatives to animal models. In 2013, the European Union completely banned animal testing in the cosmetics industry [7].

Recently, in vitro models that represent the physiologically and functionally relevant environment of human organs/tissues have been developed as an alternative to conventional animal models [8,9]. An in vitro model, by implementing similar functions and characteristics of human organs/tissues, can be used as a platform to test the performance and side effects of new drugs and cosmetics [10,11,12]. Moreover, since these are manufactured using human cells, they can overcome the issue of species barrier [13]. In vitro models can accelerate cell maturation and represent the function of native organs/tissues by implementing physical and mechanical properties such as microfluidic flow, pulse, and electrical stimulation of human organs/tissues [14,15,16,17]. To date, various in vitro models of the eyes [18], kidneys [9], skin [19], heart [20], and liver [21] have been actively developed. This study provides a review of recent bioprinting technologies and bioinks and their applications in the fabrication of in vitro models.

## 2. Commercialized In Vitro Models

### 2.1. Market Analysis of In Vitro Models

The in vitro model platform market is a nascent one (due to which the market analysis differs per each research institution) and one with a high compounded annual growth rate (CAGR). The total in vitro model market size is estimated to be USD 40–50 million (CAGR: 36.6%), with liver-on-a-chip (CAGR: 43.5%), kidney-on-a-chip (CAGR: 38.1%), lung-on-a-chip (CAGR: 30.8%), and heart-on-a-chip (CAGR 34.0%) representing the main market segments [22].

The market size of the in vitro model by region is in the order of North America > Europe > Asia. As of 2020, the North American market is estimated at USD 19 million (50.0%, CAGR 43.5%), the European market at USD 7.9 million (20.8%, CAGR 38.1%), and the Asian market at USD 5.5 million (14.4%, CAGR 30.8%) [22].

### 2.2. Companies and In Vitro Models in the Market

Several companies are active in the market of in vitro tissue models, using varying technological methods. The majority of the companies use microfluidic technology, and others provide physical or biochemical tissue environments with 3D printing technology or bioink. The list of the companies is summarized in Table 1.

Mimetas (founders: Jos Joore, Paul Vulto, and Thomas Hankemeier), is one of the leading companies in the in vitro model market. The core technology of this company provides over 100 devices in one platform, which is a very high number for an in vitro platform. The major product, OrganoPlate^TM^, is a 96-independent cell culture platform based on a 384-well-plate. This platform does not include the 3D bioprinting technology, but provides a 3D cell culture environment and offers compatibility with the existing analysis equipment.

Emulate (founder: Donald Ingber), which is based on a highly developed technology, provides various in vitro models, accessories, analysis services, and protocols. Their device contains a cyclic stretch and pneumatic controller for mimicking the environment of the tissue. Kidneys, liver, intestine, and lungs are the main target tissues, and the tissue platform is designed to provide a culture module. Imaging can also be conducted using their imaging adapter.

CN Bio (founder: Linda G Griffith), the liver in vitro model-based company, provides a liver platform, NASH (non-alcoholic fatty liver) model, and toxicity testing services. The multi-well cell culture plate was specially designed for a 3D culture environment. Their in vitro model contains a membrane-based 3D environment and fluid circulation even though their platform is based on Transwell^®^.

TissUse (founder: Uwe Marx), a Germany-based company, developed an in vitro model platform that can emulate biological environments such as the intestine, lung, skin, or liver. In addition, their platform is designed for the multi-organ model. Four organs were demonstrated on one platform over 28 days with a single device control.

Organovo (founders: Gaber Forgac, Keith Murphy), a NASDAQ listed company, is the most famous in the 3D bioprinting field, with collaborations with several global pharmaceutical and cosmetic companies. Organovo focuses on developing 3D human tissues based on their patented 3D printing system NovoGen Bioprinter^®^. Based on this 3D technology, their platform provides a strong advantage as a result of mimicking the physical environment of the tissue.

### 2.3. Technologies Required in the Development of In Vitro Models

**Figure 1:**
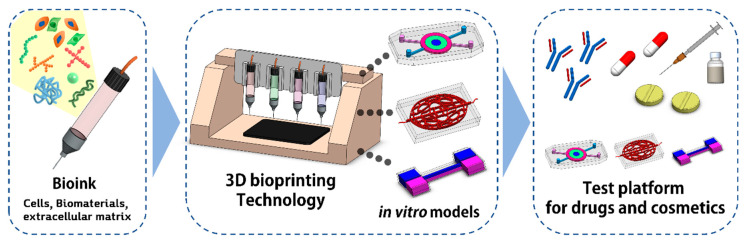
Manufacture of in vitro test models with bioinks and bioprinting.

To fabricate an in vitro model similar to a human tissue/organ, it is essential to create an intrinsic environment and architecture that offers efficient physical and chemical simulation, and the complex composition of various cells. Recent advances in 3D bioprinting technology have facilitated the development of functional tissues through the organization of various cells and biomaterials within physiologically relevant environments (Figure 1) [28,29,30]. Various 3D bioprinting techniques have been developed to fabricate 3D tissue constructs, including light-assisted, droplet-based, and extrusion-based printing systems that use computer-aided design (CAD) and computer-aided manufacturing (CAM) [10].

Bioink, one of the key elements of 3D bioprinting technology, is a cell-encapsulating material for bioprinting [11]. Most bioinks are made of hydrogel, which serves to improve the precision of bioprinting and the viability of cells. To build a 3D tissue structure with high fidelity, the bioink should provide adequate printability (e.g., viscosity, mechanical properties, and cross-linking) [11]. Depending on the printing method, bioinks may have photocurable properties or deposition abilities, which can produce high-precision 3D structures. Notably, bioink also provides an extracellular matrix for the printed cells, affecting cell proliferation, differentiation, and maturation, creating organ/tissue analogues with biofunctionality [30]. It is important to ensure the excellent printability and suitable biological properties of bioinks. The biofabrication window is based on the concept of correlation between the printability and biological properties of bioinks [31,32]. Bioinks with high printability generally have a high viscosity or crosslinking density, which can lead to reduced biological properties. Conversely, bioinks with good biofunctionality (i.e., excellent cell proliferation, differentiation, and maturation) can have low printability. The 3D tissue structure needs to maintain its architecture to secure the biological activity of the inner cells. Thus, the selection of bioink and the characteristics of the biofabrication window should be carefully considered.

## 3. Technologies for Bioprinting

Bioprinting has now evolved into an effective technology for preparing complex biological structures with biomaterials owing to the advancement of precise cell and ECM positioning. Bioprinting technology can be divided into three different methods: Micro-extrusion, droplet-based, and laser-assisted printing methods (Table 2).

### 3.1. Micro-Extrusion Printing Method

Micro-extrusion is the most commonly used method for printing 3D biological structures, wherein the print is essentially deposited on the substrate by a micro-extrusion head. In this printing, physical forces such as pneumatic or mechanical pressure can be used to selectively distribute biomaterials and cells to desired locations through nozzles and needles [33]. Heterogeneous and complex structures can be prepared by applying a micro-extrusion-based system equipped with multiple printing heads containing different cells/bioinks [34,35]. When using multiple heads, parameters such as nozzle position and spacing, printing speed, blowing force, and the nozzle diameter must be considered. In addition, sufficient viscosity of the bioink is required to maintain the shape of the structure. Although bioinks of varying viscosities can be deposited through micro-extrusion, those with higher viscosity are useful in preventing the collapse of printed structures and ensuring high-resolution printing.

### 3.2. Droplet-Based Printing Method

The droplet-based printing method allows a controlled volume (droplet) of the cell suspension liquid to be delivered to a predefined location. When the liquid passes through the printing nozzle, water droplets can be created from the microbubble formation using electric heating nozzles [36], piezoelectric actuators [37], acoustic actuators [38], and magnetic fields [39]. The electric heating printing method has a high printing speed and low cost, but the cells are exposed to heat, and the uneven droplet size is a disadvantage to reckon [33]. Printing with piezoelectric actuators can solve the aforementioned problems of electric heating, but the 15–25 kHz frequency used for piezoelectric actuators can cause cell membrane damage and lysis [40]. Droplet printing with the acoustic method uses a gentle acoustic field generated by an acoustic actuator from a bioink pool. Using this method, cells are not exposed to stress, however, acoustic fields are unstable, leading to poor printing results [38]. Droplets can also be printed using magnetic fields. A micro-valve module consists of a solenoid coil and a plunger, which blocks the bioink flow before applying the magnetic field. By applying voltage to the solenoid, a magnetic field is produced, and the plunger goes up for bioink extrusion. The droplet size can be controlled by adjusting the voltage [38].

Droplet-based printing can deliver cell droplets with much more accurate resolution than micro-extrusion printing, but large-scale biological structures cannot be printed. Despite the drawback, droplet-based bioprinting is likely to narrow down complex biological structures owing to its advantages of high-resolution droplet printing.

### 3.3. Laser-Assisted Printing Method

The laser-assisted printing system applies a laser-guided front transfer to prepare biological structures [41]. This laser-assisted printing can overcome several limitations associated with the micro-extrusion printing and droplet-based printing methods. For example, laser-assisted printing allows for droplet printing of the highest resolution owing to laser interference. The typical mechanism of laser-assisted printing is as follows: (1) The laser is focused onto a laser-absorbing support layer, the so-called ribbon (2) pulse of the laser hits the cell-laden hydrogel which is beneath the ribbon, and (3) cell droplets are printed onto the receiving substrate [42,43]. The resolution of laser-assisted printing is affected by several factors, such as the laser power, the design of the biological layer, and the interference between the ribbon and the reception. Therefore, laser-assisted printing offers the highest resolution, albeit with the need to adjust many factors, similar to other printing methods.

Stereolithography (SLA) is another type of laser-assisted printing method. The basic mechanism of SLA is to coagulate a liquid photopolymer by photopolymerization with a laser. Ultraviolet (UV), infrared (IR), or visible light is commonly used in the polymerization process. Laser pulses can solidify materials and produce solidified structures in reservoirs, which are combinations of bio-inks, cells, and photoinitiators. Finally, the 3D patterned solidified layers can be stacked to prepare 3D biological structures [44].

## 4. Technologies for Bioinks

Bioinks enable the fabrication of 3D cell structures, which have appropriate mechanical and rheological properties to maintain their structural stability. They also provide a suitable extracellular matrix to induce cell adhesion, proliferation, and differentiation after fabrication [45,46]. Among the several types of bioinks, water-soluble polymers known as hydrogels are attractive candidate materials for building cell-laden 3D structures by bioprinting technologies due to their relatively good biocompatibility and also biodegradability. Their high water content can provide a promising environment to encapsulate cells with freely exchanging nutrients, oxygen, and other supplements to maintain their viability and functionality [47,48,49]. Natural polymers are comprised of short repeating units that can induce non-covalent bonding (such as hydrogen bonding and π–π interactions). Due to this reversible interaction between inter and intra polymer chains, they usually show a shear-thinning behavior [50]. Typically, a mixture of polymer solution as bioink and desired cells to load cross-links or polymerizes by relatively cell-friendly reagent and condition [51,52,53,54]. Appropriate materials should be carefully chosen for the desired purpose, since each material for the hydrogel bioink has intrinsic characteristics. Blended polymers have been used to complement the characteristics of each polymer material to improve printability and mechanical, physicochemical, and biological properties of the bioink [55]. In particular, photo-cross-linkable polymers (e.g., PEGDA (poly(ethylene glycol) diacrylate) and GELMA (gelatin methacrylate)) are broadly used as solidifiers, and contribute to the solidification of blended bioinks using UV light [56,57]. Moreover, several additives (e.g., graphene oxides, hydroxyapatite, nano-cellulose) are added as supplements into polymer bioinks to improve their specific functionality (e.g., fidelity, differentiation) [58,59,60]. In this section, we described the use of polymer-based hydrogel materials as bioinks (Table 3).

### 4.1. Natural Polymers

#### 4.1.1. Alginate

Alginate is refined from brown seaweed, which is one of the most preferable natural hydrogels owing to its low toxicity, relatively low price, and applicability for various printing techniques [61,62]. In particular, the alginate solution can easily solidify upon mixing with divalent ions (e.g., Ca^2+^, Sr^2+^, and Ba^2+^) without the need for chemical reagents or the production of byproducts during cross-linking [41,63]. Cell-laden alginate based bioinks with the calcium solution were utilized to fabricate 3D structures by ionic cross-linking before or after printing [64,65,66]. Usually, alginate has been blended with other polymers to ensure its biological functionality [67,68,69]. Another approach is the introduction of bioactive molecules (e.g., peptides) into the alginate backbone before printing to improve its functionality [70,71]. In addition, to improve mechanical properties and structural stability, chemical cross-linking, such as methacrylation and thiolation, is also used [65,72].

#### 4.1.2. Gelatin

Gelatin is derived from collagen via acidic (type A) or basic (type B) hydrolysis, which is relatively easy to obtain in large quantities from animal sources (e.g., bones, tendons, or skins) compared to pure collagen [73]. Gelatin has a thermo-reversible gelation behavior against the surrounding temperature, which is particularly attractive as a bioink. Due to this property, the cell-laden gelatin can easily build up the desired 3D structure by regulating the temperature and concentration [74,75]. In addition, gelatin has cell responsive properties such as the RGD peptide for cell binding motif and matrix metalloproteinase (MMP) recognition sequences for degradation [76,77]. However, while gelatin is advantageous for bioprinting, it offers insufficient structural stability due to its low mechanical strength and temperature sensitivity. Chemical cross-linking (e.g., methacrylation, resulting in cross-linking via UV) can overcome these limitations and support fabricated features for a long time under various physiological conditions [78,79,80].

#### 4.1.3. Hyaluronic Acid

Hyaluronic acid, abundant in cartilage, connective tissues, and synovial fluid in our body, has excellent rheological and biochemical properties, and is an attractive material for bioink [81,82]. In particular, hyaluronic acid has been used for cartilage tissue regeneration, since it has the potential to induce chondrogenic differentiation due to the CD44 interaction with laden cells [83,84]. The use of hyaluronic acid as a bioink requires chemical modifications and/or mixing with other polymer materials, resulting in solidification of the fabricated 3D structure. Typical chemical modifications of hyaluronic acid for use as bioinks are methacrylation and thiolation to solidify the printed polymer solution [85,86]. In addition, hyaluronic acid has been mixed with other polymer materials (e.g., gelatin and β-cyclodextrin) to overcome the limitations of a single hyaluronic acid application [86,87].

#### 4.1.4. Silk Fibroin

Silk fibroin, as a biomaterial from several animal sources, has shown promising characteristics due to its superior mechanical properties and biocompatibility [88]. Although it has attractive properties as a biomaterial, there is a limitation to its use as a bioink due to the β-sheet formation by shear stress during printing [89]. To overcome this limitation, some supplements (e.g., glycerol, silica) and/or polymer materials (e.g., alginate, PEG) are added to silk fibroin in order to improve its viscoelastic and physical properties for printing [90,91,92]. Sibce silk fibroin exhibits a lack of interaction with typical adherent cells [93], other polymers (e.g., collagen, dECM) have been mixed with silk fibroin to increase its cellular activity and tissue formation capability [94,95].

#### 4.1.5. Collagen

Collagen, an abundant component in the body (approximately 25% of the total dry weight of mammals), has extremely good biological properties (e.g., cellular interaction and functional expression) [96,97]. Therefore, numerous tissue engineering and drug delivery applications have been reported for collagen [98,99]. However, pure collagen as a bioink has intrinsic limitations such as a lack of suitable mechanical properties and structural stability after bioprinting due to slow cross-linking by a shift of temperature to 37 °C [100]. To overcome this limitation, collagen has been incorporated with a different type of polymer (e.g., hyaluronic acid, alginate) [101,102]. Another promising approach for collagen as a bioink is UV cross-linking to solidify bioprinting structures via methacrylation [103].

#### 4.1.6. Fibrin

Fibrin, an essential protein involved in blood clotting and wound healing, has been utilized as a scaffold for tissue engineering due to its suitable biological properties (e.g., cell adhesion) by fibrinogen and thrombin mixing under suitable physiological conditions [104,105]. Fibrin has also been employed as a protein carrier for long-term drug delivery through its heparin-binding domains and growth factor affinity [106,107]. However, fibrin has insufficient mechanical strength and long-term instability in cells [108]. To overcome these drawbacks, fibrin is generally incorporated with other polymer materials such as gelatin, alginate, silk fibroin, and collagen to improve its mechanical properties and printability [109,110].

#### 4.1.7. Decellularized Extracellular Matrix (dECM)

Decellularized extracellular matrix (dECM), with cells removed by chemical reagents, physical and mechanical processes, can mimic the target tissue-specific environment with an original chemical composition and structural intricacy [111,112,113]. Although dECM can generate a 3D structure via a thermogelling mechanism, the sole use of dECM is difficult due to its low mechanical stability. As a result, the solubilized dECM is generally blended with other polymers or printed together with other structural supporting materials [92,114]. The dECM as a bioink has the potential to provide an understanding of how cells work in native tissue mimetic ECM with regard to cellular activity and tissue regeneration.

#### 4.1.8. Agarose

Agarose is generally used in the biochemical analysis for DNA and protein separation during electrophoresis. It has also been broadly utilized as a hydrogel material for biomedical applications, due to its biocompatibility, abundance, and simple gelation behavior resulting from temperature shifts [115,116]. To improve its printability and/or interaction with cells, agarose has been mixed with other bioactive polymers and molecules (e.g., collagen, fibrin, alginate) [117,118]. Nevertheless, the mechanical properties of agarose are not sufficient for long-term cell culture and in vivo application. To overcome these intrinsic properties, agarose has been blended with other materials or introduced via an additional cross-linking mechanism [119].

### 4.2. Synthetic Polymers

#### 4.2.1. Poly(ethylene glycol)

Poly(ethylene glycol) (PEG) is typically regarded as a relatively safe synthetic polymer, for which physical and chemical properties such as the chain length and structure can be easily controlled. It also has strong mechanical properties, non-cytotoxicity (depending on molecular weight), and non-immunogenicity as a biomaterial. However, PEG on its own cannot form a hydrogel structure with a shift in temperature or ionic cross-linking, similar to natural polymers. In addition, there is no interaction between PEG and cells to induce adhesion and other cellular activities. Therefore, PEG needs to be conjugated with functional groups (e.g., methacrylation) and/or accompany other functional polymer materials [120,121]. Pluronic^®^ F127 (PF127), a commercial block copolymer consisting of PEG and poly(propylene glycol) (PPG), has a thermo-reversible gelation behavior, resulting in having it utilized more than pure PEG as a bioink for tissue regeneration [122,123,124].

#### 4.2.2. Polysiloxane

Silicone, a common name for polysiloxane, is an elastic synthetic polymer that has been extensively utilized in the clinical field owing to its biocompatibility and mechanical durability [125]. Polysiloxane can easily engineer processing and casting at a high molecular weight and fabricate solid structures by simply mixing with a curing agent (e.g., platinum) [126]. Similar to other polymers, silicone can also be applied to a UV-curable system to form a hydrogel structure via methacrylation or thiolation. Such a structure cures relatively fast and shows low toxicity, and is suitable for biomedical applications such as bioink. Thus, polysiloxane can easily regulate photo-cross-linking reactions and fabricate 3D structures with good surface properties and fidelity [127,128].

## 5. Application to In Vitro Models

### 5.1. Respiratory System

The respiratory system consists of specialized parts for breathing to deliver oxygen to the blood. This system directly interfaces with the external environment, such as air. In addition, the respiratory tract is one of the representative organs that develop local defensive barriers to prevent the inflow of foreign substances. Therefore, the development of an in vitro model of the human respiratory system is essential to explore advanced medicine, particularly for the treatment of respiratory diseases.

**Figure 2:**
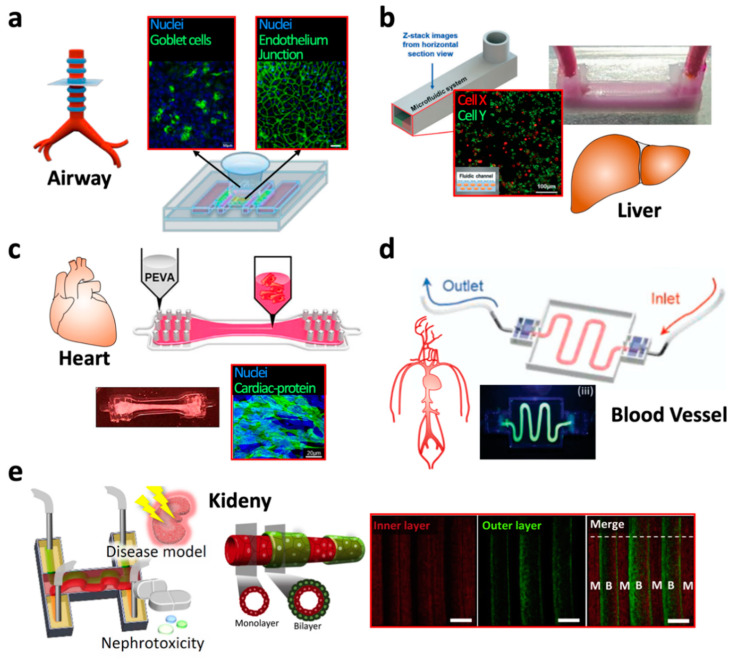
The 3D bioprinted in vitro models. (**a**) Airway-on-a-chip. The red-lined inset images indicate the formations of (left) epithelium with functional goblet cells and (right) endothelium in the chip. Adapted from Park et al. [129]. (**b**) Liver-on-a-chip. The red-lined inset image indicates a monolayer of red-dyed cells onto a bioink containing green-dyed cells. This arrangement was applied in the co-culture of endothelial cells (monolayer on the top) and hepatocytes (encapsulated in the bioink at the lower part). Adapted from Lee et al. [34]. (**c**) Bioprinted cardiac muscle. The red-lined inset image indicates the maturation of the cardiac muscle in the system. Adapted from Das et al. [20]. (**d**) Perfuable blood vessel-on-a-chip. Adapted from Gao et al. [130]. (**e**) Renal proximal tubule-on-a-chip. The red-lined inset images show a proximal tubule bilayered with renal epithelium (outer layer, green) and endothelium (inner layer, red). Adapted from Singh et al. [9].

To recapitulate the airway tissue structure consisting of an epithelium sitting on a basement of the vascular network, an epithelium-assembled vascular bed system was 3D bioprinted (Figure 2a) [129]. In this model, a cylindrical container with a porous bottom was covered with a tracheal mucosa decellularized extracellular matrix (tmdECM) to accelerate epithelial differentiation. In addition, 3D bioprinting was applied to a construct containing endothelial cells and fibroblasts mixed with tmdECM to induce vascular network formation. The epithelium monolayered container was then assembled into the construct of the vascular network. The resulting bioprinted airway-on-a-chip showed a more tighter junction formation and more mucus secretion as compared to the other airway-on-a-chip printed with collagen, which has been widely used as a matrix for 3D cell culture. Consequently, the airway-on-a-chip biorpinted with tmdECM was a better representative of the physiological features of the defense system of the airway epithelial barrier.

### 5.2. Digestive System

The digestive system, including many internal organs of the human body, is the main tract influenced by drugs administered through the oral route. In particular, the liver is a representative organ responsible for metabolism and detoxification. Therefore, in new drug development processes, the liver is frequently a subject of investigation to understand how the drug is metabolized and the resultant hepatotoxic effect.

As the liver environment includes multiple types of cells with different functions, Lee et al. applied a multi-material printing process to construct an in vitro liver model composed of various kinds of cells in a chip structure (Figure 2b). By alternately depositing different materials on demand, the entire in vitro liver model can be fabricated through a one-step process. The housing was fabricated with polycaprolactone, and liver tissue was printed with HepG2-laden collagen bioink and HUVEC-laden gelatin bioink. The bioprinted liver model showed higher hepatocyte viability and albumin/urea secretion levels [34].

### 5.3. Cardiovascular System

The cardiovascular system is responsible for transporting nutrients, gases, hormones, cytokines, and cells through a blood stream. In this system, as the heart and vascular/lymphatic vessels are connected in a closed loop, the continuous and periodic contraction of cardiac muscle induces pressure to drive blood circulation. Therefore, many soluble agents and drugs are injected intravenously to deliver the substances to the target site through the transportation system of the body. Otherwise, drugs are administered orally, are metabolized, and the byproducts circulate through the vessels. Hence, the interaction between the cardiovascular system and the drug plays a crucial role in pharmacokinetic and pharmacodynamic studies. In addition, as the cardiovascular system is constantly exposed to circulating drugs, the cardiotoxicity evaluation is an essential process in new drug development. Thus, an in vitro model of the cardiovascular system is highly valuable for establishing a reliable drug testing platform.

To monitor the contraction of the cardiac muscle cultured in vitro, a flexible and biocompatible strain gauge was 3D printed as the bottom substrate of a system containing multi-chambers. In this system, cardiac muscle monolayers were cultured on the strain gauges that exhibited a periodic contraction following differentiation and maturation. The contractile force readout was collected in a real-time manner [16,131]. In addition, Das et al. demonstrated the promising effect of a heart-derived decellularized ECM (hdECM) bioink on accelerating the maturation of cardiac muscle tissue constructs in vitro (Figure 2c) [20]. Using a 3D bioprinting process, an in vitro model of the cardiac muscle was constructed to allow fixation of the cell-laden hdECM hydrogel on poly(ethylene/vinyl acetate) anchors. The bioprinted cardiac muscle tissue hung on the anchors showed continuous compaction over time due to the stretch of muscle cells during tissue maturation. The resulting chip exhibited a higher level of differentiation markers of cardiac muscle following the use of the hdECM hydrogel bioink, compared to the use of collagen.

In the 3D bioprinting of the in vitro vessel model, a co-axial nozzle system presented the construction of vessels with a complete transportation function (Figure 2d) [130]. Via the application of a water-soluble Pluronic F-127-based hydrogel to a core nozzle and an endothelial cell-laden vessel-derived dECM bioink to a shell nozzle, a hollow tubule structure can be extruded in a hydraulic reservoir. The bioprinted vessel showed endothelial monolayer formation on the luminal surface. In addition, the resulting vessel exhibited pump-driven fluid circulation, low permeability without leakage, and functionality as an endothelium barrier for circulating cells, such as platelets and leukocytes. Thus, the applicability of 3D bioprinting has been demonstrated for the construction of an in vitro cardiac vessel model that allows the investigation of circulation, as well.

### 5.4. Renal System

The renal system includes urinary organs and is responsible for the elimination of waste from the body. In particular, kidneys consist of highly congested vascular capillaries in the nephron and glomerulus, which are specialized units for the filtration and removal of toxins. Therefore, the circulating byproducts of drug metabolism frequently affect the nephron filtration system, and the renal proximal tubule becomes a primary target for drug-induced toxicity. Hence, the development of an in vitro model of renal tissue has been a remarkable achievement that drew significant attention.

Homan et al. first employed a 3D bioprinting process to create the intricately winding structure of the proximal tubule [132]. A winding path was printed with a PF-127 hydrogel on a pre-cast gelatin-based hydrogel, and then the entire chamber was embedded with a gelatin-based hydrogel. As the PF-127 hydrogel turns into a liquid state from the solid state at a low temperature, such as 4 °C, while the gelatin hydrogel forms a solid at the chilled temperature, the PF-127-printed path could be eliminated via a light negative pressure, leaving a hollow channel in the low-temperature condition. Finally, the renal proximal tubular cells were coated on the luminal surface of the channel. The bioprinted renal tissue model showed morphological and molecular maturation, and also demonstrated destruction of the renal epithelium barrier following the treatment with cyclosporine A, a well-known drug of nephrotoxicity. In addition, Lin et al. exhibited the bioprinting of vessel tubules alongside the printed proximal tubule structure. The co-bioprinted tubules showed mass transportation across the luminal surfaces, as seen in the native nephron [133]. Furthermore, the application of the kidney-derived decellularized ECM (kdECM) bioink to print proximal tubules demonstrated the superior efficacy of kdECM in inducing functionalities of the renal epithelium barrier, compared to that of collagen (Figure 2e) [9].

## 6. Conclusions and Future Aspects

Significant progress has been made in the development of in vitro models using bioprinting technology. Various types of 3D bioprinting technologies, including droplet-based, extrusion-based, and light-assisted bioprinting, can be utilized to fabricate in vitro models that mimic the structural and functional features of human organs and tissues by positioning cells and bioinks in a spatiotemporal manner. Bioinks, mainly made of hydrogel, are responsible for the resolution of bioprinting and affect the precision and mechanical strength of a bioprinted structure [10,32]. Bioinks also encapsulate cells, providing a microenvironment for cell survival, proliferation, differentiation, and maturation, and preserve cells from negative exogenous factors that occur during the printing process [31]. To create an in vitro model that exhibits biological functions which are similar to those of human tissues/organs, it is important to replicate the physical, chemical, and biological properties of native tissues/organs [14,15,16,17].

Thus, the biochemical cues of bioink should be properly modulated. The cellular microenvironment composed of ECM and soluble factors (e.g., cytokines and growth factors) guides cell behavior, function, and fate, and has a great influence on tissue regeneration [134]. Since the composition of the microenvironment differs based on the tissue type and location, modulating the specific composition of the bioink for each tissue is necessary. The decellularization bioink, which has been actively studied in recent years, has the advantage of using the unique ECM environment of each tissue. However, there are challenges that need to be addressed, such as the low mechanical properties and poor deposition ability of the decellularized bioink [135]. Recently, attempts to overcome these limitations have been made by introducing an additional cross-linking agent [136] or a new printing method [137] to the dECM bioink.

Securing both tissue regeneration ability and printing ability in bioink simultaneously is difficult. Tailoring the printing ability requires changing the concentration or cross-linking density, whereas increasing these parameters can result in a lower biocompatibility. Therefore, the development of an advanced bioprinting technology is needed to break out of this traditional biofabrication window.

Recent advances in bioprinting modalities have offered the possibility of addressing these challenges. The microfluidic-based bioprinting technology enables simultaneous dispensing of various bioinks and cross-linking agents, ensuring a high shape fidelity of bioinks [138]. The FRESH technique facilitates the manufacture of 3D volumetric structures by dispensing a low-viscosity bioink into a gel-based bath [139].

The development of advanced bioinks is also breaking the boundaries of biofabrication windows. Bioinks with low initial viscosity can tackle low shape fidelity and printability through various stimuli-responsive hydrogels.

Thermo-responsive hydrogels undergo sol-gel conversion due to the network alteration in response to temperature changes. Various thermo-responsive bioinks such as gelatin and PF-127 play a major role in the deposition ability of bioinks [140]. These are added to bioink compositions to improve their deposition capacity, or to print supports with sacrificial bioinks that help create architecture with overhang structures.

A shear-responsive bioink is characterized by crosslinking hydrogels using shear stress. In silk fibroin, the β-sheet transition promotes a self-assembly behavior and hydrogel formation. Since the β-sheet transition can be induced by shear stress, the application of silk fibroin properties to bioink has been developed [141]. The advantage of shear-responsive bioinks is that no additional crosslinking agents are required. Thus, they are cell friendly and their mechanical strength and viscosity can be controlled spatiotemporally [142].

Photo-responsive hydrogels have the property of changing polymer chains when stimulated by light. In bioink development, these hydrogels are mainly used for cross-linking purposes. Photo-responsive bioinks can secure a wide range of biofabrication windows through changes in light irradiation time and photoinitiator concentration. In particular, they can overcome the traditional biofabrication window when used with bioprinting modalities with added light irradiation. Performing UV crosslinking while printing photocurable bioink through the nozzle can improve the printability of bioink with low viscosity [143].

Nevertheless, current, single, component hydrogel-based bioinks simultaneously rarely meet major requirements, including adequate printability and high cellular functionality. An effective method to secure a wider biofabrication window may be the development of multi-component bioinks using the respective advantages of single-component bioinks (e.g., deposition ability, tissue specificity, cross-linking method, viscosity, etc.) [52].

Through the advanced bioprinting modalities and bioinks, it is possible to manufacture external structures that can provide physical simulation, and to create a chemical and biological microenvironment. Recent advanced bioprinted in vitro models effectively reflect the characteristics of normal and diseased tissue models, and they can therefore be used to understand the disease mechanism of pathogens and determine the dose and efficacy of therapeutic agents [8,11,137,144]. In addition, the various advantages of bioprinting make it useful for the human-on-a-chip development that implements various organs/tissues (such as heart, liver, lung, intestine, and bone) as one model [144,145]. Through this type of multi-organ modeling, complex drug metabolism, which cannot be simulated on a single-organ model, can be realized as in an in vivo human environment.

## Figures and Tables

**Table 1:** Summary of the companies that provide in vitro models and their technical production methods with strengths and weaknesses.

Company	Product Name	Country	Strength	Weakness	Scientific Ref
Mimetas	OrganoPlate^TM^	Netherlands	Multiple assays in one plate, able to apply various cell types (neurons, hepatocytes, endothelial cell, etc.)	High cell density (10^8^ cells/cm^3^), organ specific biochemical environment, cannot provide a one directional flow	[23]
Emulate	Liver-Chip	USA	Cyclic stretch and pneumatic controller to mimic the environment of the tissue	No 3D environment	[24]
CN Bio	PhysioMimix ^TM^	UK	Membrane based 3D environment, transwell plate but has a fluid circulation	Less number of assays in one plate	[25]
TissUse	HUMIMIC CHIP	Germany	Multi-organ platform	Less number of assays in one plate	[26]
Organovo	ExVive3D TM, NovoGen Bioprinter^®^	USA	3D bioprinting technology (mimic the physical environment of the tissue well)	No microfluidic condition	[27]

**Table 2:** Characteristics of various bioprinting methods.

Bioprinting Methods	Characteristics
Micro-extrusion	🞄Most commonly produced by the micro-extrusion method that prints directly onto the substrate using a micro-extrusion head. 🞄By using physical force, biological materials and cells are selectively sprayed to the desired location through a nozzle.
Droplet-based	🞄Print a controlled volume (fine droplets) of bioink containing cells at the location to be printed. 🞄Droplet-based bioprinting can be categorized in thermal, piezoelectric, magnetic-assisted, and acoustic bioprinting.
Laser-assisted	🞄In photo-curing printing, biological structures are patterned and printed by the laser-guided forward transfer. 🞄Stereolithography generally uses a solidification method of liquid photopolymers by laser-induced photopolymerization at ultraviolet, infrared, or visible wavelengths.

**Table 3:** Natural and synthetic polymer candidates as bioinks in biomedical applications.

Type	Material	Advantage	Disadvantage	Typical Cross-Linking
Natural	Alginate	Simple gelation Good stability	Less cell interaction Less biodegradation	Ionic
	Gelatin	Low antigenicity Low cost	Less stable Low mechanical properties	UV
	Hyaluronic acid	Good cell interaction Good angiogenesis	Rapid degradation Poor mechanical stability	UV
	Silk fibroin	Slow degradation Good mechanical properties	Allergic response Less cell interaction	Physical
	Collagen	Good cell interaction	Less stability Low mechanical properties	Thermal
	Fibrin	Good angiogenesis Fast gelation	Poor mechanical stability Easily clogs	Enzymatic
	Decellularized extracellular matrix (dECM)	Similar to native ECM composition and structure	Low shape fidelity Low mechanical properties	Thermal
	Agarose	Simple gelation	Less stability Low mechanical properties	Thermal
Synthetic	Poly ethylene glycol (PEG)	Reproducibility Easy chemical modification	Low cell interaction Poor mechanical strength	UV
	Polysiloxane	Good mechanical properties Slow degradation	Low cell interaction Relatively expensive	UV

## Data Availability

The data presented in this study are available on request from the corresponding author.
